# Effects of Dietary *Terminalia chebula* Extract on Growth Performance, Immune Function, Antioxidant Capacity, and Intestinal Health of Broilers

**DOI:** 10.3390/ani14050746

**Published:** 2024-02-28

**Authors:** Ying Cheng, Shida Liu, Fang Wang, Tao Wang, Lichen Yin, Jiashun Chen, Chenxing Fu

**Affiliations:** 1 Animal Nutritional Genome and Germplasm Innovation Research Center, College of Animal Science and Technology, Hunan Agricultural University, Changsha 410128, China; 15350383010@163.com (Y.C.); lsdhnkf@163.com (S.L.); wf980313@163.com (F.W.); 15700726316@163.com (L.Y.); 2 Institute of Subtropical Agriculture, Chinese Academy of Sciences, Changsha 410125, China; wttrue@163.com

**Keywords:** *Terminalia chebula* extract, immune function, antioxidant capacity, intestinal health, yellow-feathered broiler

## Abstract

**Simple Summary:**

High-density intensive farming accelerates the incidence and transmission risk of various infectious diseases in poultry. The development and application of medicinal herbs, probiotics, and other feed additives have gathered a lot of attention in the context of the total ban on antibiotic feed additives and healthy breeding. This study investigated the effects of dietary *Terminalia chebula* extract (TCE), a plant extract, on growth performance, immune function, antioxidant capacity, and intestinal health in yellow-feathered broilers. Our findings demonstrated that TCE could improve broilers’ immune function, antioxidant capacity, intestinal health, and growth performance. The mechanism for the beneficial effect of TCE supplementation was not limited to the enhancement of the immune response and antioxidant capacity, as there was also an improvement in intestinal health due to TCE enhancing beneficial bacteria, promoting intestinal short-chain fatty acid secretion, and improving intestinal morphology. This improvement in intestinal health ultimately resulted in the improved health status of the yellow-feathered broilers. These findings provide a basis and reference for *Terminalia chebula* extract as an effective feed additive in broiler breeding.

**Abstract:**

*Terminalia chebula* extract (TCE) has many physiological functions and is potentially helpful in maintaining poultry health, but its specific effect on the growth of broilers is not yet known. This research investigated the effects of dietary *Terminalia chebula* extract (TCE) supplementation on growth performance, immune function, antioxidant capacity, and intestinal health in yellow-feathered broilers. A total of 288 one-day-old yellow-feathered broilers were divided into four treatment groups (72 broilers/group), each with six replicates of 12 broilers. The broilers were given a basal diet of corn–soybean meal supplemented with 0 (control), 200, 400, and 600 mg/kg TCE for 56 d. The results demonstrated that, compared with the basal diet, the addition of TCE significantly increased (linear and quadratic, *p* < 0.05) the final body weight and overall weight gain and performance and decreased (linear and quadratic, *p* < 0.05) the feed-to-gain ratio in the overall period. Dietary TCE increased (linear, *p* < 0.05) the levels of IgM, IL-4, and IL-10 and decreased (linear and quadratic, *p* < 0.05) the level of IL-6 in the serum. Dietary TCE increased (linear and quadratic, *p* < 0.05) the levels of IL-2 and IL-4, decreased (linear and quadratic, *p* < 0.05) the level of IL-1β, and decreased (linear, *p* < 0.05) the level of IL-6 in the liver. Dietary TCE increased (linear and quadratic, *p* < 0.05) the level of IgM and IL-10, increased (linear, *p* < 0.05) the level of IgG, and decreased (linear and quadratic, *p* < 0.05) the levels of IL-1β and IL-6 in the spleen. Supplementation with TCE linearly and quadratically increased (*p* < 0.05) the catalase, superoxide dismutase, glutathione peroxidase, and total antioxidant capacity activities while decreasing (*p* < 0.05) the malonic dialdehyde concentrations in the serum, liver, and spleen. TCE-containing diets for broilers resulted in a higher (linear and quadratic, *p* < 0.05) villus height, a higher (linear and quadratic, *p* < 0.05) ratio of villus height to crypt depth, and a lower (linear and quadratic, *p* < 0.05) crypt depth compared with the basal diet. TCE significantly increased (linear, *p* < 0.05) the acetic and butyric acid concentrations and decreased (quadratic, *p* < 0.05) the isovaleric acid concentration. Bacteroidaceae and Bacteroides, which regulate the richness and diversity of microorganisms, were more abundant and contained when TCE was added to the diet. In conclusion, these findings demonstrate that supplementing broilers with TCE could boost their immune function, antioxidant capacity, and gut health, improving their growth performance; they could also provide a reference for future research on TCE.

## 1. Introduction

At present, the high-density intensive feeding model makes broilers who are highly sensitive to various stimuli prone to oxidative stress and immune damage, as well as increasing the incidence and transmission risk of various infectious diseases [1,2,3]. During oxidative stress, free radicals increase in the body, and the body is in a peroxidation state, which will cause oxidative damage to cells, tissues, and organs [4]. Immune cells are very sensitive to oxidative stress, and their immune function decreases after being stimulated by excessive free radicals, which in turn affects the function of the immune system [5]. The gut is the first line of defense against external stimuli and is in contact with the outside world. When the intestinal barrier is damaged or the flora is dysfunctional, the gut needs to secrete immunoglobulin and cytokines to resist the damage of external pathogens to the body [6]. Therefore, the antioxidant capacity, immune system, and intestinal health of broilers jointly affect body health.

Antibiotics can effectively reduce the harm to animals caused by intensive farming methods, improving the growth of livestock and poultry [7]. However, misuse has also emerged, leading to bacterial resistance, environmental contamination, and antimicrobial residues in food of animal origin [8,9,10]. Several countries have drafted legislation and regulations to prohibit the use of antibiotics in recent years. China has also published a similar notice outlawing antibiotics in feed starting 1 July 2020. Therefore, it is necessary to find alternatives that can lower feed costs, promote animal growth, and lower the risk of disease prevention and control, such as antimicrobial peptides, probiotics, Chinese herbal feed additives, and plant extracts [11,12,13]. Plant extracts can promote livestock and poultry growth and have anti-inflammatory, antioxidant, and other effects. Therefore, the application of plant extracts in actual production has a promising future [14,15].

*Terminalia chebula*, a traditional Chinese medicinal herb, is a member of the genus *Terminalia* and has pharmacological properties such as hypoglycemic, antibacterial, and antioxidant effects [16,17,18]. Tannins, phenolic acids, flavonoids, and polysaccharides are the principal active ingredients of *Terminalia chebula* extract (TCE) [19,20,21]. According to several in vivo and in vitro studies, the main biological functions of TCE include anti-inflammatory, antioxidant, bacteriostatic, and immunomodulatory effects [22,23,24]. According to Lee et al. [25], *Terminalia chebula* fructus water extract significantly decreased serum lipid peroxidation induced by copper sulfate, scavenged 1,1-diphenyl-2-picrylhydrazyl, and decreased nitric oxide production, cyclooxygenase-2, and inducible nitric oxide synthase expression in macrophage. Another study found that the chebulic acid of *Terminalia chebula* Retz. increased intracellular glutathione levels, heme oxygenase-1 activity, and expression of the nuclear factor erythroid 2-related factor 2 (Nrf2) target gene and was protective against *tert*-butyl hydroperoxide-induced oxidative stress and reactive oxygen species in HepG2 cells [26]. An extract from *Terminalia chebula* Retz. fruit showed antimicrobial activity through the inhibition of bacterial, inflammatory, and autoimmune diseases [27]. According to Gaire et al. [28], the ability of TCE to inhibit nitric oxide accumulation by 76.2% at 10 μg/mL suggests that it has anti-inflammatory properties. There is also a study that found that *Terminalia chebula*’s ethyl acetate extract can affect the intestinal flora of mice and alleviate intestinal damage in mice [29].

Serum contains a variety of nutrients and a large number of immunoglobulins, growth factors, and hormones. The spleen is the largest peripheral immune organ in poultry and an important organ in the immune response [30]. The liver is the main location of the body’s antioxidant function. The liver also has some immune functions and contains a large number of immune cells [31,32]. The small intestine is one of the important organs for poultry in digesting and absorbing nutrients, and it is also a congenital barrier to keeping the internal environment stable [33,34]. The immune and antioxidant status of serum, the spleen, and the liver, as well as intestinal health, can reflect the growth and development of broilers. To our knowledge, no studies on the application of TCE in poultry production have been conducted. Therefore, this study investigated the effects of supplemental TCE on the growth performance, immune function, antioxidant capacity, and gut health of yellow-feathered broiler chickens.

## 2. Materials and Methods

### 2.1. Animals, Experimental Design, and Management

A total of 288 male yellow-feathered chickens that were one day old and of a similar body weight (BW) of 31.13 ± 0.13 g were purchased from a commercial hatchery (Hunan Xiangjia Animal Husbandry Co., Ltd., Changde, China) and randomized into four treatment groups (72 broilers/group), each having six replicates of 12 broilers. The chickens were administered basal diets containing 0 mg/kg TCE (control) or 200, 400, or 600 mg/kg TCE (TCE-supplemented groups) [35,36]. The experimental design is shown in Table 1. The TCE used in this study was supplied by Wuxi Zhengda Biology Co., Ltd., Wuxi, China. The pulp of *Terminalia chebula* is extracted by water, filtered to remove the residue, concentrated, and then spray dried to produce the finished TCE. The main bioactive compounds of the TCE were tannins (46%), polysaccharides (14.2%), and flavonoids (3.16%). The birds were raised in environmentally controlled wire cages (110 cm × 50 cm × 60 cm). Water and feed were provided ad libitum with continuous lighting. The room temperature was kept at 33 ± 1 °C for the first 3 d, decreasing by 2 °C to 3 °C every week until the room temperature dropped to 22 °C, and then remained at 22 °C until the end of the 56-day experiment. In order to meet the nutrient requirements of broilers, the basal diet was created in two phases (1–28 d and 28–56 d) in accordance with the Nutrition Research Council (1994) and the Feeding Standard of Chicken (NY/T 33-2004, China) [37]. In Table 2, the composition of the basal diet and its nutritional levels are displayed.

### 2.2. Sample Collection

One broiler per replicate (six replicates per treatment group) with a body weight most similar to the average body weight of the replicate was randomly selected on day 56. Broilers were weighed and slaughtered by cervical dislocation for sample collection after fasting for 12 h [38,39]. Blood was collected from the wing vein before slaughter using 5 mL non-anticoagulant vacuum tubes (Shandong Aosite Medical Equipment Co., Ltd., Heze, China). These blood samples were stored for 30 min before being separated into serum samples by centrifuging them for 10 min at 4 °C at 3000× *g* based on the methods of Zhang and Piao [40]. Prior to analysis, serum samples were stored at −20 °C. Euthanasia and necropsy were then performed on these birds. The liver and spleen samples were immediately collected. To observe the morphology of the intestine, segments of about 2 cm in the middle of the duodenum, jejunum, and ileum were preserved in formalin solution. Cecal chyme was collected aseptically using sterile tubes, rapidly frozen in liquid nitrogen with all samples, and stored at −80 °C for further analysis. Following Song et al. [41], prior to analysis, the samples of the livers and spleens were homogenized with ice-cold 0.9% saline at a weight (g)-to-volume (mL) ratio of 1:9 and then centrifuged for 15 min at 3000× *g* at 4 °C. After collecting and applying the supernatant, the total protein of the liver and spleen tissue homogenates was determined with diagnostic kits (Nanjing Jiancheng Bioengineering Institute, Nanjing, China) according to the manufacturer’s instructions.

### 2.3. Growth Performance

In each replicate cage, the broilers were weighed on days 1, 28, and 56 after a 12 h fast. The amount of feed consumed from 1 to 28 d and from 28 to 56 d in each cage was recorded, and the average daily gain (ADG), average daily feed intake (ADFI), and feed-to-gain ratio (F/G) were calculated for each cage.

ADG (g) = [final BW (g) − initial BW (g)]/days

ADFI (g) = total feed consumed (g)/days

F/G = total feed consumption (g)/weight gain (g)

### 2.4. Immune Function

Chicken ELISA kits (Huamei Biological Engineering Research Institute, Wuhan, China) were used to determine the serum, spleen, and liver immunoglobulin A (IgA), IgG, IgM, interleukin 1β (IL-1β), IL-2, IL-4, IL-6, and IL-10 concentrations according to the manufacturer’s instructions. Principle: (a) This assay employs the competitive inhibition enzyme immunoassay technique. The microtiter plate provided in this kit is pre-coated with IgA/IgG/IgM. Standards or samples are added to the appropriate microtiter plate wells with a Horseradish Peroxidase (HRP)-conjugated antibody preparation specific for /IgG/IgM. The competitive inhibition reaction is launched between the pre-coated /IgG/IgM and /IgG/IgM in the samples. A substrate solution is added to the wells, and the color develops inversely with the amount of /IgG/IgM in the sample. (b) The color development is stopped, and the intensity of the color is measured. This assay employs the quantitative sandwich enzyme immunoassay technique. An antibody specific for IL-1β/IL-2/IL-4/IL-6/IL-10 is pre-coated on a microplate. Standards and samples are pipetted into the wells, and any IL-1β/IL-2/IL-4/IL-6/IL-10 present is bound by the immobilized antibody. After removing any unbound substances, a biotin-conjugated antibody specific for IL-1β/IL-2/IL-4/IL-6/IL-10 is added to the wells. After washing, avidin-conjugated Horseradish Peroxidase (HRP) is added to the wells. Following a wash to remove any unbound avidin–enzyme reagent, a substrate solution is added to the wells, and the color develops in proportion to the amount of IL-1β/IL-2/IL-4/IL-6/IL-10 bound in the initial step. The color development is stopped, and the intensity of the color is measured.

### 2.5. Antioxidant Capacity

The manufacturer’s instructions were followed when measuring the total antioxidant capacity (T-AOC), malonic dialdehyde (MDA) level, and superoxide dismutase (SOD), catalase (CAT), and glutathione peroxidase (GSH-Px) activities in the serum, liver, and spleen using diagnostic kits (Nanjing Jiancheng Bioengineering Institute, Nanjing, China). Principle: (a) ABTS is oxidized to green ABTS^+^ under the action of appropriate oxidants, and the production of ABTS^+^ is inhibited in the presence of antioxidants. The total antioxidant capacity of the sample can be determined by measuring the absorbance of ABTS^+^ at 405 nm or 734 nm. Trolox, a VE analog, has a similar antioxidant capacity to VE and is used as a reference for the total antioxidant capacity of other antioxidants. (b) MDA in the degradation products of lipid peroxide can be condensed with thiobarbituric acid (TBA). A red product is formed, with a maximum absorption peak at 532 nm. (c) The decomposition reaction of hydrogen peroxide (H_2_O_2_) by CAT can be quickly stopped by adding ammonium molybdate, and the remaining H_2_O_2_ reacts with ammonium molybdate to produce a light-yellow complex. The activity of CAT can be calculated by measuring its change at 405 nm. (d) GSH-PX can promote the reaction of H_2_O_2_ with reduced glutathione to produce H_2_O and oxidized glutathione. The activity of glutathione peroxidase can be expressed by the rate of its enzymatic reaction. Then, the activity of the enzyme can be found. (e) See Figure S1.

### 2.6. Intestinal Morphology

The fixed duodenum, jejunum, and ileum segments were removed from a 10% neutral buffered formalin solution and embedded in paraffin; then, they were prepared for sectioning at a 5 μm thickness and stained with hematoxylin and eosin, as described by Chang and Yu [42]. A computer-assisted morphometric system under a biological microscope (MSHOT ML31) was used to measure the average intestinal villus height (VH), crypt depth (CD), and the ratio of villus height to crypt depth (VH/CD) on intestinal sections with integral morphology.

### 2.7. Short-Chain Fatty Acid Analysis

In accordance with Wang et al. [43], 1 g of cecal chyme was mixed with 6 mL of sterile water and centrifuged at 12,000× *g* at 4 °C for 15 min. Then, 900 μL of supernatant was mixed with 100 μL of 25% meta-phosphoric acid solution and centrifuged at 12,000× *g* at 4 °C for 15 min. Afterwards, a 0.45 mm syringe filter was used to filter 600 μL of supernatant, and the short-chain fatty acid (SCFA) concentrations were determined via gas chromatography. The run conditions for the gas chromatography procedure were as follows: A DB-FFAP column was selected as the chromatographic column, with a specification of 30 m × 250 μm × 0.25 μm. The carrier gas was high-purity nitrogen (99.999%), and the flow rate was 0.8 mL/min. The auxiliary gas was high-purity hydrogen (99.999%), the detector FID temperature was 280 °C, the inlet temperature was 250 °C, the split ratio was 50:1, and the injection volume was 1 μL. Programmed temperature rise: the initial temperature was 60 °C, and the temperature was raised to 220 °C at a rate of 20 °C/min and held for 1 min [44].

### 2.8. 16S rRNA Sequencing of Microflora in Cecum Contents

Microbial genomic DNA was extracted from cecum digesta using QIAamp Fast DNA Stool Mini Kits (Qiagen, Hilden, Germany) following the manufacturer’s instructions. The V3–V4 hypervariable regions of bacterial 16S rRNA were amplified using primers 338F (5′-ACTCCTACGGGAGGCAGCA-3′) and 806R (5′-GGACTACHVGGGTWTCTAAT-3′), respectively. The PCR products were extracted via 2% agarose gel electrophoresis and purified using a DNA gel extraction kit (Axygen Biosciences, Union City, CA, USA). They were then quantified with a QuantusTM fluorometer (Promega, Madison, WI, USA) and sequenced using an Illumina platform (San Diego, CA, USA) (Hiseq or Miseq). The data were analyzed on the Majorbio Cloud online platform (www.majorbio.com) accessed on 16 February 2023.

Based on overlap relationships, Flash version 1.2.11 was used to splice PE reads from the Illumina sequencing. Sequences were quality-controlled and filtered. Meanwhile, the samples were distinguished, and OTU clustering analysis (Uparse version 11, http://www.drive5.com/uparse/, accessed on 13 March 2023) and species taxonomic analysis were performed. Based on the findings of the clustering analysis, alpha diversity analysis (Mothur version 1.30.2, https://www.mothur.org/wiki/Download_mothur, accessed on 13 March 2023) was performed. Based on taxonomic information, the community structure was statistically analyzed (Qiime version 1.9.1, http://qiime.org/install/index.html, accessed on 13 March 2023) at different taxonomic levels. Species showing variations in abundance in various microbial community groupings (samples) based on community abundance in the sample were determined using rigorous statistical methods. The grouped samples were subjected to an inter-group similarity analysis to test the differences’ significance. Then, using a supervised learning approach, further linear discriminations were carried out on the groupings of the sample divisions.

### 2.9. Statistical Analysis

Data analysis was carried out with the replicate as the experimental unit of the growth performance data and the individual broiler per cage as the experimental unit of the other data. The effect of dietary TCE levels was assessed using linear and quadratic orthogonal polynomial contrasts. Multiple comparisons were used to compare the differences between the groups. The GLM procedure of the Statistical Package for Social Sciences 27.0 (SPSS Inc., Chicago, IL, USA) was used in the analysis. The results were presented as means and the pooled standard error of the mean (SEM). Values of *p* < 0.05 were regarded as statistically significant.

## 3. Results

### 3.1. Growth Performance

As shown in Table 3, the addition of dietary TCE linearly and quadratically increased (*p* < 0.05) the final BW of the broilers at 56 days of age compared to the control group (Figure S2A). There was a linear and quadratic increase (*p* < 0.05) in the ADG in the finisher and the overall periods and a linear and quadratic decrease (*p* < 0.05) in F/G in the overall period with increasing dietary TCE levels compared to the control (Figure S2B,C). The ADFI of the treatment group with 200 mg/kg TCE was significantly higher (*p* < 0.05) than that of the control group and treatment group with 400 mg/kg TCE.

### 3.2. Immune Function

According to Table 4, the supplementation of TCE linearly increased (*p* < 0.05) the IgM and IL-4 contents in the serum (Figure S3A,B). Compared to the control, in the treatment groups supplemented with 400 and 600 mg/kg TCE, there was a significant decrease (linear and quadratic, *p* < 0.05) in the serum IL-6 content (Figure S3C), and supplementation of the diet with 400 mg/kg TCE significantly increased (linear, *p* < 0.05) the serum IL-10 content (Figure S3D). Dietary supplementation with 600 mg/kg TCE significantly increased (linear and quadratic, *p* < 0.05) the levels of IL-2 and IL-4 and significantly decreased (linear and quadratic, *p* < 0.05) the levels of IL-1β in the liver compared to the control (Figure S4A–C). There was a significant decrease (linear, *p* < 0.05) in the IL-6 content in the liver when TCE was added at 200 and 600 mg/kg (Figure S4D). In the spleen, the IgM content was significantly increased (linear and quadratic, *p* < 0.05) when TCE was added at 600 mg/kg compared to the control group (Figure S5A). The addition of TCE to the diet significantly increased (linear, *p* < 0.05) the IgG level and significantly decreased (linear and quadratic, *p* < 0.05) the IL-6 level (Figure S5B,C), with an optimal effect at 400 mg/kg. Compared to the control, dietary additions of 400 and 600 mg/kg TCE significantly increased (linear and quadratic, *p* < 0.05) the IL-10 level as well as significantly decreased (linear and quadratic, *p* < 0.05) the IL-1β level (Figure S5D,E).

### 3.3. Antioxidant Capacity

Table 5 shows that the supplementation of TCE in the diet affected the antioxidant capacity. In comparison with the control group, serum T-AOC was significantly increased (linear and quadratic, *p* < 0.05) when the diets were supplemented with 400 and 600 mg/kg TCE, and GSH-Px activity was significantly increased (linear and quadratic, *p* < 0.05) when the diets were supplemented with 600 mg/kg TCE (Figure S6A,B). TCE supplementation increased (*p* < 0.05) liver T-AOC linearly and quadratically (Figure S6C). Splenic CAT and GSH-Px activities were significantly increased (linear and quadratic, *p* < 0.05) in the treatment group with the addition of 600 mg/kg TCE compared to the control group (Figure S6D,E). The SOD activity in the spleen was increased (*p* < 0.05) linearly (Figure S6F).

### 3.4. Intestinal Morphology

**Figure 1:**
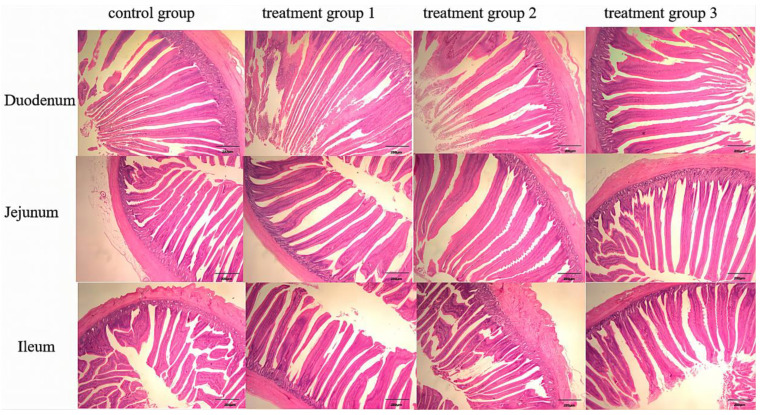
Microscopic sections of broilers’ intestinal morphology.

As shown in Figure 1 and Table 6, compared to the control diet, duodenal VH was increased (linear and quadratic, *p* < 0.05) in the group supplemented with 400 mg/kg TCE (Figure S7A). The duodenal VH/CD was increased (linear and quadratic, *p* < 0.05), and CD was decreased (linear and quadratic, *p* < 0.05) in the groups supplemented with 400 and 600 mg/kg TCE (Figure S7B,C). With the addition of 400 mg/kg TCE, the VH and VH/CD of the ileum were increased (quadratic, *p* < 0.05) (Figure S7D,E).

### 3.5. SCFA Analysis

Table 7 displays a linear increase (*p* < 0.05) in the acetic acid and butyric acid concentrations in response to the intake of TCE in the diet (Figure S8A,B). On the other hand, compared to the other groups, a significant decrease (quadratic, *p* < 0.05) in the isovaleric acid content was observed in the group supplemented with 200 mg/kg TCE (Figure S8C).

### 3.6. 16S rRNA Sequencing of Microflora in Cecum Contents

**Figure 2:**
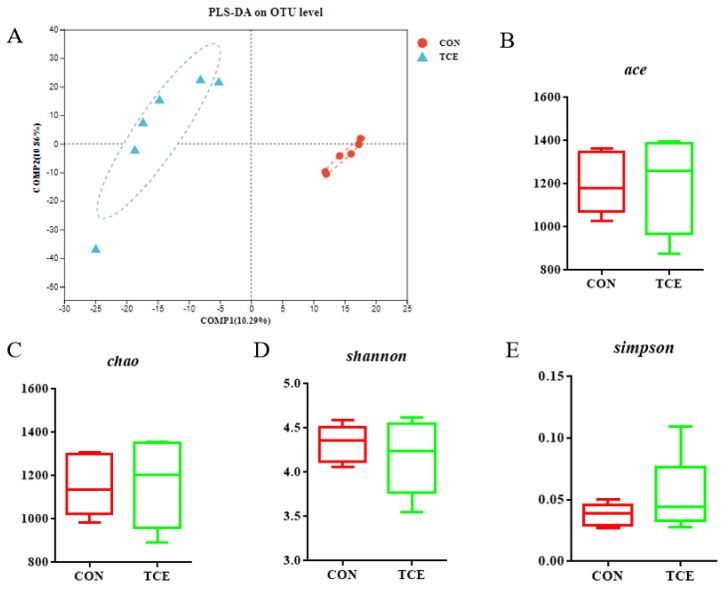
Partial least squares discriminant analysis (PLS-DA) of the Bray–Curtis dissimilarities for the cecal samples (**A**). Ace, Chao, Shannon, and Simpson indexes of the gut microbiota (**B**–**E**). CON, basal diet; TCE, basal diet supplemented with 600 mg/kg *Terminalia chebula* extract.

The control and 600 mg/kg TCE treatment groups were subjected to 16S rRNA sequencing of the cecal microflora to examine group differences after considering the effects of 56-day basal and experimental diets on growth performance. In the present study, 1,657,981 high-quality sequences were retrieved from 12 intestinal samples, averaging 138,165 sequences per sample. Partial least squares discriminant analysis clearly distinguished between CON and TCE, indicating microbial compositional differences between them (Figure 2A). As shown in Figure 2B–E, the groups showed no differences in four alpha diversity estimators (Ace, Chao, Simpson, and Shannon).

**Figure 3:**
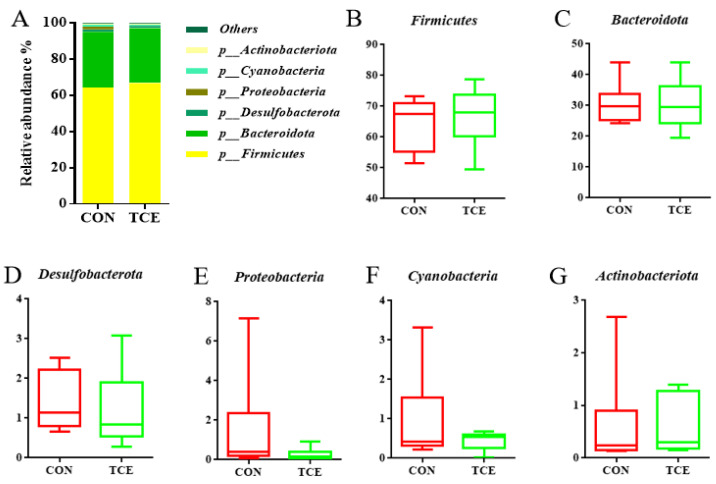
Microbial composition at the phylum level (**A**). Top 6 at the phylum level (**B**–**G**). CON, basal diet; TCE, basal diet supplemented with 600 mg/kg *Terminalia chebula* extract.

**Figure 4:**
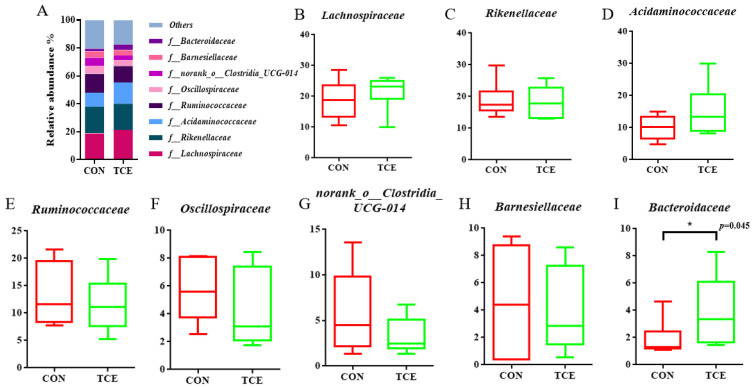
Microbial composition at the family level (**A**). Top 8 at the family level (**B**–**I**). CON, basal diet; TCE, basal diet supplemented with 600 mg/kg *Terminalia chebula* extract.

**Figure 5:**
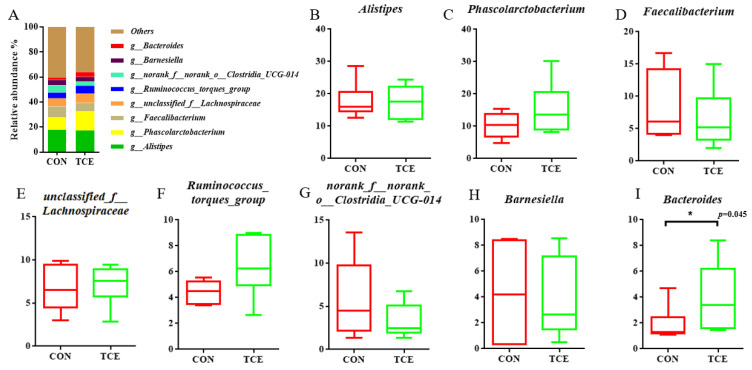
Microbial composition at the genus level (**A**). Top 8 at the genus level (**B**–**I**). CON, basal diet; TCE, basal diet supplemented with 600 mg/kg *Terminalia chebula* extract.

The top six phyla of the cecal microbiota were the Firmicutes, the Bacteroidota, the Desulfobacterota, the Proteobacteria, the Cyanobacteria, and the Actinobacteriota (Figure 3A). There were no significant differences among the top six phyla, as shown in Figure 3B–G. Lachnospiraceae and Rikenellaceae were the two families that were most prevalent at the family level across all samples (Figure 4A), followed by Acidaminococcaceae, Ruminococcaceae, Oscillospiraceae, norank_o__Clostridia_UCG-014, Barnesiellaceae, and Bacteroidaceae. Compared to the CON group, the abundance of Bacteroidaceae was higher (*p* < 0.05) in the TCE dietary treatment (Figure 4B–I). Intestinal flora that predominated in all samples at the genus level included Alistipes, Phascolarctobacterium, Faecalibacterium, unclassified_f__Lachnospiraceae, Ruminococcus_torques_group, norank_f__norank_o__Clostridia_UCG-014, Barnesiella, and Bacteroides (Figure 5A). As shown in Figure 5B–I, TCE supplementation significantly increased (*p* < 0.05) the abundance of Bacteroides.

### 3.7. Correlation Analysis

**Figure 6:**
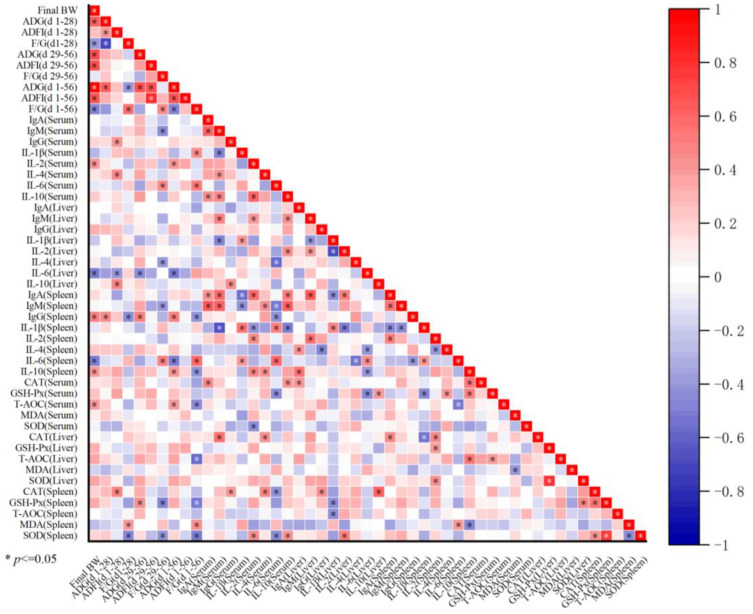
Pearson’s correlation analysis between growth performance, antioxidant factors, and immune factors affected by dietary treatment. The chromaticity of a color indicates the strength of the correlation. Red represents a significant positive correlation (*p* < 0.05), blue represents a significantly negative correlation (*p* < 0.05), and white shows that the correlation was not significant (*p* > 0.05).

Pearson correlation analysis was performed on growth performance, immune factors, and oxidative parameters (Figure 6). Taking the growth performance of the whole stage as an example, overall weight gain was positively correlated with IL-2 and T-AOC in serum, IgG and IL-10 in the spleen, and negatively correlated with IL-6 in the liver and spleen. The overall F/G was positively correlated with serum IL-1β and IL-6, spleen IL-6 and MDA, and negatively correlated with spleen IgG, IL-10, GSH-Px, SOD, serum, and liver T-AOC. Moreover, correlations between immune factors and oxidation parameters were also found. Overall, these results indicated that the change in growth performance was related to changes in immune function and antioxidant capacity.

## 4. Discussion

In recent years, with people’s attention on food safety, the use of Chinese herbal medicine and plant extract feed additives in animal production has grown. Herbal *Terminalia chebula* has analgesic, antibacterial, and anticancer functions [45,46,47]. Recent research suggests that TCE contains flavonoids, tannins, phenolic acids, and other active ingredients [48,49] and performs physiological activities that are bacteriostatic, antioxidant, and anti-inflammatory [28,50,51]. Zhang et al. [52] reported that in broilers challenged with aflatoxin B1, Chinese gallnut tannic acid could increase the ADG and ADFI, decrease the plasma MDA concentration, increase plasma total SOD, GSH-Px, and T-AOC activities, increase the ileal VH, and decrease the jejunal CD. Additionally, according to Prihambodo et al. [53], feeding broilers flavonoids increased the ADG, decreased F/G, decreased cholesterol and malondialdehyde concentrations, and increased SOD activity, the duodenal VH and VH/CD, and the jejunal VH/CD. Plant polysaccharides increase antioxidant and immune capacity, improve broiler intestinal morphology, regulate intestinal health, and enhance the growth performance of broilers [54,55]. These studies have shown that the effects of dietary supplementation with plant extracts with similar active ingredients and functions on growth performance are related, at least in part, to enhancing immune and antioxidant capacity and controlling the intestinal flora [56,57]. In the case of tannins, they can condense with proteins through hydrophobic or hydrogen bonding to form stable tannin–protein complexes, which can play a role in intestinal astringency and maintain intestinal health [58]. When the body is subjected to oxidative stress, tannins can block Nrf2 ubiquitination, and the phenolic hydroxyl group in the periphery of the tannin molecule eliminates reactive oxygen species by releasing hydrogen atoms and grabbing electrons, restoring homeostasis [59,60]. When the inflammatory signal is transmitted downstream and the nuclear factor-κB inhibitor protein (IκB) is phosphorylated and modified by the nuclear factor-κB inhibitor protein kinase, NF-κB is released, and hydrolyzed tannins block the translocation of NF-κB to the nucleus, inhibiting the expression of pro-inflammatory factors and alleviating the inflammation [61]. Tannins can inhibit bacteria by disrupting the integrity of bacterial cell walls and cell membranes or by robbing microorganisms of essential nutrients for metabolism [62,63]. This study discovered that the ADG increased in both the finisher and overall periods when the TCE concentration increased, while F/G decreased in the overall period. This is closely related to the fact that active substances such as tannic acid in TCE can improve the body’s immune performance, antioxidant performance, and intestinal health. These previous results support the conclusion of this study that dietary TCE may boost immune and antioxidant functions, enhance intestinal structure and function, and thus promote growth performance. In addition, the results of our correlation analysis also confirm this conclusion.

IgA, IgM, and IgG, which are produced by plasma cells that differentiate from B cells, are critical for maintaining a balance between pro- and anti-inflammatory responses and for regulating many immune functions [64,65]. Thl and Th2 cells are essential mediators and participants in cellular and humoral immune responses, and their balance is a vital signal for measuring inflammatory responses [66]. IL-lβ, IL-2, IL-4, IL-6, IL-10, and other cytokines are secreted by both Th1 and Th2 cells to sustain normal immune function [67]. Among them, IL-4 and IL-10, which can reduce inflammation, are called anti-inflammatory factors, while IL-6 and IL-1β, which can promote inflammation, are called pro-inflammatory factors [68,69]. IL-2, a T-cell growth factor produced by T cells or T-cell lines, is involved in hematopoietic functions and the regulation of white blood cell activity. In the current study, adding TCE to the diet resulted in higher serum, liver, and spleen IL-2, IL-4, and IL-10 concentrations. It also resulted in lower serum, liver, and spleen IL-1β and IL-6 concentrations. According to one study [50], chebulanin and chebulagic acid from *T. chebula* can reduce the expression of the inflammatory cytokines IL-6 and IL-8. The inflammatory cytokines IL-1β, IL-6, and tumor necrosis factor-α were shown to be present at lower levels in mouse serum treated with a standardized *Terminalia chebula* extract, according to Seo et al. [70]. These findings support those of our experiment and show that TCE supplementation can increase the anti-inflammatory factors and reduce the pro-inflammatory factors, thus enhancing broiler immunity and resistance to inflammation. The production of IL-1β and IL-6 is related to the NF-κB signaling pathway, which is one of the important pathways regulating the inflammatory response in the body [71]. Research has found that Terminalia chebula Retz. extract inhibits the phosphorylation of IĸBα and the nuclear translocation of p65 and p50 in vitro [36]. Haghani et al. [47] reported that TCE could inhibit the protein expression of the NF-κB signaling pathway. Liu et al. [72] found that TCE decreased the protein expression levels of Toll-like receptor 4, myeloid differentiation factor 88, p-IκBα, and p-p65 and the nuclear translocation of p65 in vitro. These results suggest that TCE may inhibit the expression of inflammatory factors by inhibiting the NF-κB signaling pathway. The impact of TCE on the organism’s Ig content has not been studied yet. However, previous research has demonstrated that active components of TCE, such as flavonoids and phenolic acids, may boost Ig levels and improve animal immune function [43,73]. Dietary quercetagetin increased blood IgG levels, according to Wu et al. [74], who identified the flavonol compound chemically. The levels of IgM and IgA in serum were higher in broiler chickens fed diets supplemented with 1 or 2 g/kg *Acanthopanax* polysaccharides, according to Long et al. [75]. Tian et al. [76] discovered that condensed tannin supplementation increased the immune function in cadmium-poisoned captive black goats by increasing the concentrations of IgG, IgM, and IgA. According to the current study, supplementation with TCE increased serum IgM and spleen IgM and IgG concentrations, which is consistent with the above findings. The results demonstrated that the combined action of TCE’s active ingredients could improve broilers’ immune function.

T-AOC is mostly used as a description of the coordinated activity of multiple antioxidant enzymes and related biological molecules that eliminate free radicals in specific organs [77,78]. Antioxidant capacity is linked to different antioxidant systems in the body, including enzymes and non-enzymes, which maintain the redox balance in animals [79]. Antioxidants (such as glutathione) and SOD can convert excess free radicals produced by the body into hydrogen peroxide, which can then be eliminated. Then, GSH-PX and CAT break down hydrogen peroxide into water and oxygen [80]. Among others, MDA levels are also frequently used to assess the status of lipid peroxidation. A previous study found that TCE significantly decreased MDA levels and scavenged 96% of the free radical diphenyl-1-picrylhydrazyl [28]. According to Choi et al. [81], *Terminalia chebula* water extract preconditioning improved acute liver injury induced by *tert*-butylhydroperoxide, for example, by reducing the increased MDA level, increasing the TAC and GSH contents, and increasing the decreased CAT, GSH-Px, and SOD activities. Ahmadi-Naji et al. [82] reported that treatment with the fruit extract of *Terminalia chebula* could protect against diazinon-induced oxidative stress by increasing the activities of SOD and CAT and decreasing the level of MDA. In this study, dietary TCE supplementation increased the serum, liver, and spleen CAT, SOD, and GSH-Px activities and T-AOC while decreasing the MDA concentration, indicating that TCE enhanced the antioxidant ability of the broilers. The Nrf2 signaling pathway is one of the important pathways related to oxidative stress, and it can maintain the stable state of REDOX in the body [83]. According to Jung et al. [26], chebulic acid upregulated Nrf2, glutamate-cysteine ligase catalytic subunit, glutamate-cysteine ligase modifier subunit, and heme oxygenase-1 (HO-1) expression in vitro. Zeng et al. [84] found that Terminalia chebula retzius extracts activated the Nrf2 pathway. Lin et al. [85] also found that polyphenol extracts from Terminalia chebula Retz. promoted Nrf2 nuclear translocation and enhanced Nrf2 and HO-1 expression in the nucleus. Chebulic acid increased antioxidant-responsive elements and promoted Nrf2 nuclear translocation [86]. These findings suggest that TCE can improve the content of antioxidant enzymes and the body’s antioxidant capacity by activating the Nrf2 signaling pathway.

Intestinal morphology and development are closely correlated with the ability of an animal to absorb nutrients [87]. The absorptive area of the intestine increases with VH. The higher the VH/CD, the better the digestion and absorption capacity, while the deeper the CD, the worse the digestion and absorption capacity [88]. In the current study, we discovered that the VH and VH/CD were higher in the duodenum and ileum and that the CD was lower in the duodenum of broilers supplemented with TCE rich in tannins, polysaccharides, and flavonoids. These findings are supported by earlier research. Li et al. [89] found that supplementation with dandelion tannins or soy isoflavones increased the jejunal VH and decreased the jejunal CD. Jing et al. [90] found that adding tannins to broiler diets increased VH/CD and decreased CD. Dietary astragalus polysaccharide supplementation in broilers increased the jejunal VH/CD and decreased the jejunal CD [91]. An increased jejunal VH and VH/CD may increase digestion and absorption surface area and efficiency [39]. In addition, a higher nutrient absorption surface area was found in jejuna with decreased CDs and increased VHs and VH/CDs, according to Tang et al. [92]. The current study found higher VH and VH/CD values and lower CD values in the TCE-treated groups. This suggests that TCE may enhance intestinal morphology to promote effective nutrient absorption, improve feed efficiency, and enhance broiler growth performance.

SCFAs are produced by intestinal microorganisms for the fermentation and degradation of indigestible substances and play a significant role in the host body [93,94]. They can regulate intestinal environment stability and intestinal endocrine function and inhibit pathogen growth and colonization [95,96]. In addition, intestinal microorganisms and epithelial cells use SCFAs as a major energy source [97,98]. Acetic acid is the main substrate for cholesterol synthesis, most of which is absorbed into the blood and metabolized by the liver as energy for the surrounding tissues [99]. Propionic acid is absorbed by the liver and involved in gluconeogenesis, and it inhibits cholesterol synthesis [100]. Monogastric animals may directly absorb and use butyric acid, a key source of body energy. It may also improve the intestinal barrier by affecting the proliferation and differentiation of intestinal epithelial cells [101]. In the current study, TCE increased the acetic and butyric acid concentrations while decreasing the isovaleric acid levels, and it increased the Bacteroidaceae content and Bacteroides abundance. Studies have shown that Bacteroides are involved in the production of short-chain fatty acids, especially butyric acid [102,103]. The results of this study could be explained by the proportionate increase in related bacteria that produce SCFAs.

Besides digesting and absorbing various nutrients, the gut is the main site of microbial colonization. Intestinal microorganisms assist in nutrient absorption and substance metabolism, promoting body development and maintaining host health [104]. In the body’s immune defense function, intestinal microorganisms can strengthen the gut mucosal barrier and modulate the general immunity of the host to effectively suppress the threat of pathogenic microorganisms [105,106]. There is increasing evidence that the intestinal microbiota might affect poultry production [107,108]. The Firmicutes and the Bacteroidetes are considered to be the most significant phyla in the cecal microflora of the chickens. They are believed to be closely linked to the production performance of chickens in terms of output [109]. Among them, Bacteroides can decompose complex polysaccharides, lipids, and proteins in addition to enhancing the intestinal environment and increasing the metabolic capacity of the entire intestine by producing short-chain fatty acids during intestinal metabolism to preserve intestinal homeostasis [110,111]. According to studies, Bacteroides have been implicated in intestinal inflammation and barrier dysfunction [112]. According to Lin et al. [113], quercetin supplementation increased the numbers of Bacteroides and other bacteria. Wassie et al. [114] found that supplementation with Enteromorpha polysaccharide increased the abundance of the Bacteroidetes phylum and Bacteroides genus, and the increased cecal levels of acetic, butyric, and propionic acids suggested that Enteromorpha polysaccharides enhanced the ability of the microbiota to induce SCFAs, which is in line with our results. In this study, dietary TCE treatments increased the Bacteroidaceae content and Bacteroides abundance. These findings may help to support the idea that dietary TCE can affect the quantity of probiotics in broiler ceca and regulate the abundance and diversity of microorganisms, thus regulating gut health.

## 5. Conclusions

The findings of this study show that TCE supplementation could increase the final BW and overall weight gain and decrease F/G in the overall period of broilers, inhibit the inflammatory response, enhance immune function and antioxidant capacity, and improve gut health by enhancing gut morphology, SCFAs, and the microbiome composition. TCE supplementation may also improve intestinal morphology, increase beneficial bacteria, regulate the metabolites produced by the intestinal microbiota, maintain intestinal health, and improve the growth performance of broilers. These results suggest that TCE might be used as a potential feed additive for chickens. Dietary addition of TCE can promote the growth and maintain the health of broilers, but the specific mechanism of function of TCE in the broiler organism is still unclear and needs to be further studied.

## Figures and Tables

**Table 1:** Experimental design.

Group	Diets
Control group	basal diet
Treatment group 1	basal diet + 200 mg/kg
Treatment group 2	basal diet + 400 mg/kg
Treatment group 3	basal diet + 600 mg/kg

**Table 2:** Composition and nutrient content of basal diets.

Items ^1^	Age (Days)	
	Starter	Finisher
	(1 to 28 Days)	(29 to 56 Days)
Ingredient (%)		
Corn	55.57	66.40
Wheat bran	0.60	0.50
Soybean meal	35.00	22.51
Soya oil	2.62	1.94
Fish meal	1.40	5.00
HCl-lysine	0.10	0.02
DL-methionine	0.30	0.09
Limestone	1.14	0.82
Dicalcium phosphate	1.96	1.43
Sodium chloride	0.3	0.3
Vitamin–mineral premix ^2^	1.00	1.00
Nutrient content ^3^ (%)		
ME (MJ/kg)	12.12	12.55
CP	21.18	19.00
Ca	1.00	0.90
Total P	0.74	0.70
Available P	0.45	0.45
Digestible Lys	1.24	1.03
Digestible Met	0.62	0.43

^1^ CP, crude protein; ME, metabolizable energy. ^2^ Supplied per kilogram of diet: vitamin A, 12,000 IU; vitamin D3, 2500 IU; vitamin E, 20 mg; vitamin K3, 3.0 mg; vitamin B1, 3.0 mg; vitamin B2, 8.0 mg; vitamin B6, 7.0 mg; vitamin B12, 0.03 mg; pantothenic acid, 20.0 mg; niacin, 50.0 mg; biotin, 0.1 mg; folic acid, 1.5 mg; choline chloride, 500 mg; Cu, 8.0 mg; Fe, 100 mg; Mn, 100 mg; Zn, 75.0 mg; I, 0.7 mg; Se, 0.4 mg. ^3^ The nutrient contents are calculated values.

**Table 3:** Effects of *Terminalia chebula* extract (TCE) on the growth performance of yellow-feathered broilers from day 1 to day 56 ^1^.

Parameters ^2^	TCE Level ^3^ (mg/kg)				SEM ^4^		*p*-Value	
	0	200	400	600		Anova	Linear	Quadratic
Initial BW (g)	31.17	31.04	31.08	31.22	0.130	0.869	0.581	0.723
Final BW (kg)	1.76 ^c^	1.91 ^b^	1.84 ^a^	1.86 ^a^	0.014	<0.001	0.049	0.004
Starter period, d 1 to 28								
ADG (g)	19.15	21.30	20.36	20.09	0.300	0.079	0.499	0.100
ADFI (g)	36.76	38.51	38.00	37.88	0.415	0.526	0.452	0.412
F/G (feed/gain, g/g)	1.90	1.84	1.83	1.85	0.016	0.331	0.205	0.177
Finisher period, d 29 to 56								
ADG (g)	43.21 ^b^	44.21 ^a^	44.20 ^a^	44.75 ^a^	0.152	<0.001	<0.001	<0.001
ADFI (g)	99.77 ^b^	106.54 ^a^	99.43 ^b^	103.63 ^ab^	0.902	0.006	0.591	0.683
F/G (feed/gain, g/g)	2.35	2.32	2.25	2.29	0.015	0.092	0.050	0.079
Overall, from d 1 to 56								
ADG (g)	30.82 ^c^	33.61 ^b^	32.28 ^a^	32.71 ^a^	0.249	<0.001	0.049	0.004
ADFI (g)	69.24 ^b^	72.35 ^a^	68.32 ^b^	70.34 ^ab^	0.488	0.012	0.872	0.855
F/G (feed/gain, g/g)	2.22 ^b^	2.15 ^a^	2.13 ^a^	2.15 ^a^	0.009	<0.001	0.002	<0.001

^1^ Data represent the means and pooled standard error of the mean of six replicate cages (*n* = 6). ^2^ BW, body weight; ADG, average daily gain; ADFI, average daily feed intake; F/G, feed-to-gain ratio. ^3^ Diets supplemented with 0, 200, 400, and 600 mg/kg TCE. ^4^ SEM: standard error of the mean. ^a,b,c^ Means with different superscripts within the same row mean a significant difference at *p* < 0.05.

**Table 4:** Effects of *Terminalia chebula* extract (TCE) on the immune function of yellow-feathered broilers on day 56 ^1^.

Parameters ^2^	TCE Level ^3^ (mg/kg)				SEM ^4^		*p*-Value	
	0	200	400	600		Anova	Linear	Quadratic
Serum								
IgA (g/L)	0.26	0.27	0.28	0.28	0.078	0.670	0.204	0.451
IgM (g/L)	0.69	0.70	0.78	0.77	0.017	0.103	0.028	0.086
IgG (g/L)	1.97	1.96	1.99	2.05	0.054	0.943	0.586	0.820
IL-1β (pg/mL)	76.89	68.99	58.98	62.01	3.345	0.241	0.066	0.134
IL-2 (pg/mL)	260.02	284.77	288.27	286.44	6.805	0.435	0.179	0.256
IL-4 (pg/mL)	48.18	51.28	54.76	55.29	1.261	0.142	0.021	0.068
IL-6 (pg/mL)	49.04 ^b^	46.77 ^b^	39.09 ^a^	38.20 ^a^	1.263	<0.001	<0.001	<0.001
IL-10 (pg/mL)	120.24 ^b^	133.01 ^ab^	143.37 ^a^	141.90 ^ab^	3.883	0.125	0.026	0.055
Liver								
IgA (μg/mL prot)	28.32	30.16	31.26	31.43	0.857	0.582	0.176	0.367
IgM (μg/mL prot)	85.96	86.63	89.48	88.75	1.648	0.873	0.460	0.750
IgG (μg/mL prot)	177.24	178.35	202.53	184.52	4.809	0.137	0.294	0.360
IL-1β (pg/mg prot)	12.27 ^a^	11.90 ^ab^	11.55 ^ab^	10.9 ^b^	0.182	0.057	0.005	0.021
IL-2 (pg/mg prot)	39.09 ^b^	39.88 ^ab^	41.25 ^ab^	44.43 ^a^	0.822	0.095	0.014	0.039
IL-4 (pg/mg prot)	6.70 ^b^	7.34 ^ab^	7.41 ^ab^	8.13 ^a^	0.172	0.021	0.002	0.011
IL-6 (pg/mg prot)	4.59 ^a^	4.10 ^b^	4.23 ^ab^	4.10 ^b^	0.076	0.062	0.049	0.068
IL-10 (pg/mg prot)	10.43	10.55	10.96	11.09	0.187	0.563	0.155	0.373
Spleen								
IgA (μg/mL prot)	25.00	26.98	27.07	27.04	0.759	0.745	0.371	0.549
IgM (μg/mL prot)	77.16 ^b^	77.45 ^b^	80.81 ^ab^	82.35 ^a^	0.802	0.042	0.005	0.021
IgG (ug/mL prot)	217.43 ^b^	240.19 ^a^	241.29 ^a^	238.54 ^a^	3.714	0.059	0.050	0.027
IL-1β (pg/mg prot)	13.80 ^a^	12.23 ^ab^	10.53 ^b^	10.30 ^b^	0.528	0.052	0.006	0.021
IL-2 (pg/mg prot)	37.18	37.82	38.15	37.62	0.339	0.808	0.600	0.618
IL-4 (pg/mg prot)	6.34	6.74	7.24	6.98	0.193	0.423	0.411	0.244
IL-6 (pg/mg prot)	4.10 ^a^	3.21 ^b^	3.38 ^b^	3.27 ^b^	0.103	0.002	0.008	0.002
IL-10 (pg/mg prot)	9.73 ^b^	11.11 ^ab^	11.71 ^a^	11.52 ^a^	0.288	0.052	0.017	0.019

^1^ Data represent the means and pooled standard error of the mean of six replicate cages (*n* = 6). ^2^ IgA, immunoglobulin A; IgG, immunoglobulin G; IgM, immunoglobulin M; IL-1β, interleukin-1β; IL-2, interleukin-2; IL-4, interleukin-4; IL-6, interleukin-6; IL-10, interleukin-10. ^3^ Diets supplemented with 0, 200, 400, and 600 mg/kg TCE. ^4^ SEM: standard error of the mean. ^a,b^ Means with different superscripts within the same row mean a significant difference at *p* < 0.05.

**Table 5:** Effects of *Terminalia chebula* extract (TCE) on the antioxidant activity of yellow-feathered broilers on day 56 ^1^.

Parameters ^2^	TCE Level ^3^ (mg/kg)				SEM ^4^		*p*-Value	
	0	200	400	600		Anova	Linear	Quadratic
Serum								
CAT (U/mL)	16.13	16.93	18.79	17.11	0.403	0.114	0.189	0.124
GSH-Px (U/mL)	950.83 ^b^	1048.39 ^ab^	1213.82 ^a^	1228.17 ^a^	39.343	0.021	0.002	0.009
T-AOC (U/mL)	0.49 ^b^	0.50 ^b^	0.52 ^ab^	0.55 ^a^	0.007	0.007	<0.001	0.002
MDA (nmol/mL)	2.95	2.80	2.90	3.02	0.097	0.717	0.731	0.767
SOD (U/mL)	55.99	61.36	57.04	65.32	3.060	0.902	0.399	0.688
Liver								
CAT (U/mg)	59.37	68.31	78.87	70.14	5.027	0.623	0.352	0.451
GSH-Px (U/mg)	55.94	60.51	62.48	61.80	3.079	0.892	0.490	0.728
T-AOC (U/mg)	0.57^b^	0.64 ^a^	0.64 ^a^	0.65 ^a^	0.011	0.017	0.010	0.007
MDA (nmol/mg)	0.57	0.55	0.54	0.47	0.038	0.818	0.359	0.637
SOD (U/mg)	14.98	16.29	16.20	17.76	0.530	0.342	0.081	0.225
Spleen								
CAT (U/mg)	12.10 ^b^	13.69 ^ab^	14.32 ^ab^	17.24 ^a^	0.667	0.037	0.004	0.016
GSH-Px (U/mg)	96.05 ^b^	108.62 ^b^	117.68 ^ab^	135.94 ^a^	4.585	0.008	<0.001	0.003
T-AOC (U/mg)	0.13	0.14	0.19	0.27	0.028	0.313	0.066	0.161
MDA (nmol/mg)	0.98	0.84	0.77	0.87	0.035	0.177	0.194	0.085
SOD (U/mg)	8.65	10.05	12.16	12.04	0.643	0.157	0.029	0.080

^1^ Data represent the means and pooled standard error of the mean of six replicate cages (*n* = 6). ^2^ CAT, catalase; GSH-Px, glutathione peroxidase; T-AOC, total antioxidant capacity; MDA, malondialdehyde; SOD, superoxide dismutase. ^3^ Diets supplemented with 0, 200, 400, and 600 mg/kg TCE. ^4^ SEM: standard error of the mean. ^a,b^ Means with different superscripts within the same row mean a significant difference at *p* < 0.05.

**Table 6:** Effects of *Terminalia chebula* extract (TCE) on the intestinal morphology of yellow-feathered broilers on day 56 ^1^.

Parameters ^2^	TCE Level ^3^ (mg/kg)				SEM ^4^		*p*-Value	
	0	200	400	600		Anova	Linear	Quadratic
Duodenum								
VH (μm)	826.56 ^b^	831.74 ^b^	912.78 ^a^	862.22 ^b^	9.976	0.002	0.032	0.033
CD (μm)	140.65 ^b^	132.81 ^b^	109.99 ^a^	111.07 ^a^	4.051	0.004	<0.001	0.003
VH/CD (μm: μm)	5.67 ^b^	6.14 ^b^	7.83 ^a^	7.74 ^a^	0.301	0.008	0.002	0.006
Jejunum								
VH (μm)	787.15	802.01	814.81	794.68	27.894	0.989	0.891	0.947
CD (μm)	98.13	90.99	90.63	89.06	4.137	0.886	0.469	0.734
VH/CD (μm: μm)	8.18	8.84	9.31	9.06	0.289	0579	0.236	0.373
Ileum								
VH (μm)	448.25 ^b^	545.92 ^b^	683.10 ^a^	496.95 ^b^	26.746	0.005	0.245	0.008
CD (μm)	67.61	61.92	60.54	59.95	2.636	0.750	0.312	0.543
VH/CD (μm: μm)	6.67 ^c^	8.93 ^b^	11.78 ^a^	8.47 ^bc^	0.480	<0.001	0.053	<0.001

^1^ Data represent the means and pooled standard error of the mean of six replicate cages (*n* = 6). ^2^ VH, villus height; CD, crypt depth; VH/CD, the ratio of villus height to crypt depth. ^3^ Diets supplemented with 0, 200, 400, and 600 mg/kg TCE. ^4^ SEM: standard error of the mean. ^a,b,c^ Means with different superscripts within the same row mean a significant difference at *p* < 0.05.

**Table 7:** Effects of *Terminalia chebula* extract (TCE) on the short-chain fatty acids of yellow-feathered broilers on day 56 ^1^.

Parameters ^2^	TCE Level ^3^ (mg/kg)				SEM ^4^		*p*-Value	
	0	200	400	600		Anova	Linear	Quadratic
Acetic acid (mg/g)	2.46	2.48	2.68	2.64	0.038	0.084	0.026	0.084
Propionic acid (mg/g)	0.59	0.78	0.77	0.64	0.045	0.350	0.760	0.189
Butyric acid (mg/g)	0.43	0.46	0.60	0.62	0.038	0.200	0.037	0.119
Isobutyric acid (mg/g)	0.14	0.11	0.10	0.10	0.007	0.254	0.063	0.127
Valeric acid (mg/g)	0.16	0.15	0.12	0.13	0.011	0.538	0.156	0.350
Isovaleric acid (mg/g)	0.16 ^b^	0.14 ^a^	0.15 ^ab^	0.16 ^b^	0.003	0.035	0.920	0.042
Total SCFAs (mg/g)	3.95	4.13	4.43	4.28	0.093	0.301	0.115	0.196

^1^ Data represent the means and pooled standard error of the mean of six replicate cages (*n* = 6). ^2^ SCFAs, short-chain fatty acids. ^3^ Diets supplemented with 0, 200, 400, and 600 mg/kg TCE. ^4^ SEM: standard error of the mean. ^a,b^ Means with different superscripts within the same row mean a significant difference at *p* < 0.05.

## Data Availability

The data presented in this study are available on request from the corresponding author. The availability of the data is restricted to investigators based at academic institutions.

## Supplementary Materials

The following supporting information can be downloaded at: https://www.mdpi.com/article/10.3390/ani14050746/s1, Figure S1: Principle of the determination of SOD activity; Figure S2: Significant effects of *Terminalia chebula* extract (TCE) on the growth performance of yellow-feathered broilers from day 1 to day 56; Figure S3: Significant effects of *Terminalia chebula* extract (TCE) on the immune function in serum of yellow-feathered broilers on day 56; Figure S4: Significant effects of *Terminalia chebula* extract (TCE) on the immune function in liver of yellow-feathered broilers on day 56; Figure S5: Significant effects of *Terminalia chebula* extract (TCE) on the immune function in spleen of yellow-feathered broilers on day 56; Figure S6: Significant effects of *Terminalia chebula* extract (TCE) on the antioxidant activity of yellow-feathered broilers on day 56; Figure S7: Significant effects of *Terminalia chebula* extract (TCE) on the intestinal morphology of yellow-feathered broilers on day 56; Figure S8: Significant effects of *Terminalia chebula* extract (TCE) on the short-chain fatty acids of yellow-feathered broilers on day 56.
