# Supplementary material for: Effects of Dietary Terminalia chebula Extract on Growth Performance, Immune Function, Antioxidant Capacity, and Intestinal Health of Broilers
Source: Animals (Basel). 2024 Feb 28;14(5):746. doi: 10.3390/ani14050746 (PMC10931075; doi:10.3390/ani14050746)
Supplement: Supplementary file 1 [file animals-14-00746-s001.zip › animals-2855000-supplementary.pdf]

Supplementary Material

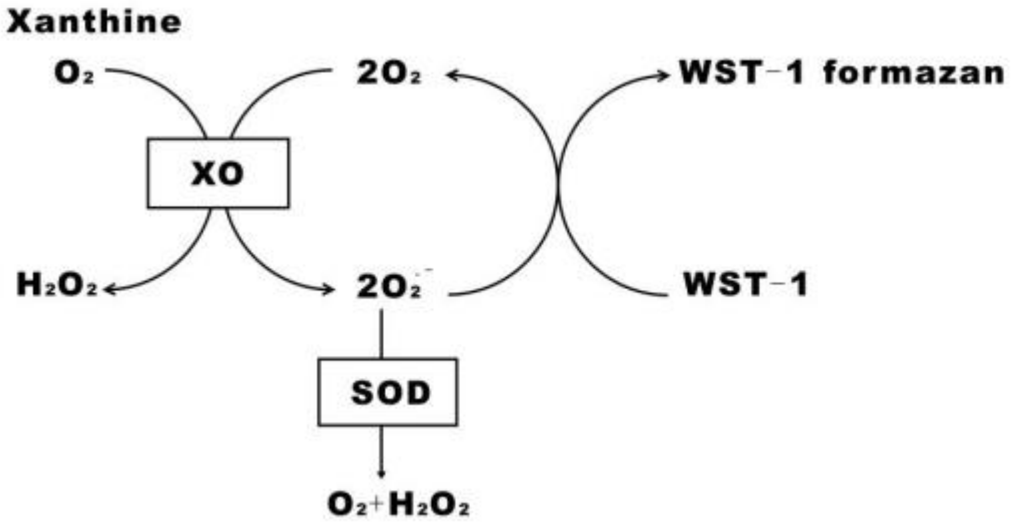

Figure S1. Principle of the determination of SOD activity.

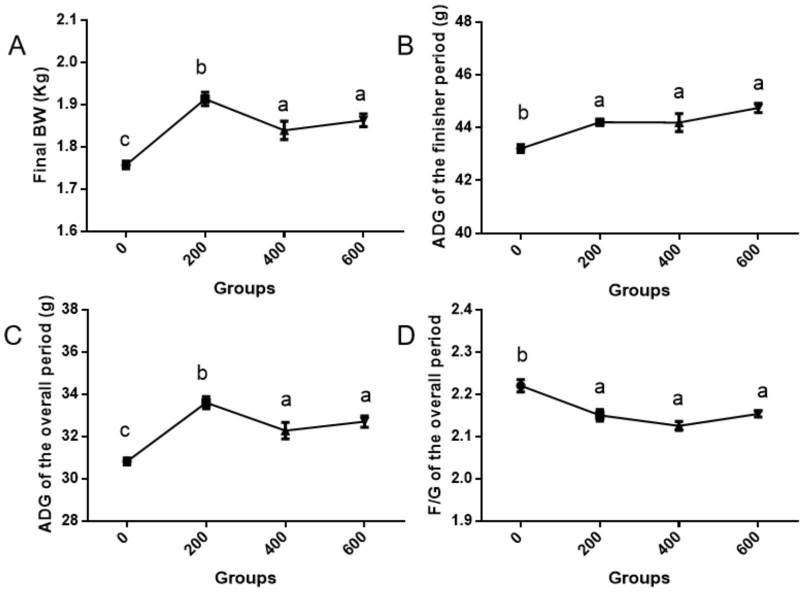

Note: Data represent the means and standard error of mean of 6 replicate cages (n = 6). BW, body weight; ADG, average daily gain; F/G, feed-to-gain ratio. Groups: The chickens were administered basal diets containing 0 mg/Kg TCE (control) or 200, 400, or 600 mg/Kg TCE (TCE-supplemented groups). a,b,c: Means with different letter within the same graph mean significant difference at $p < 0.05$.

Figure S2. Significant effects of *Terminalia chebula* extract (TCE) on the growth performance of yellow-feathered broilers from day 1 to day 56.

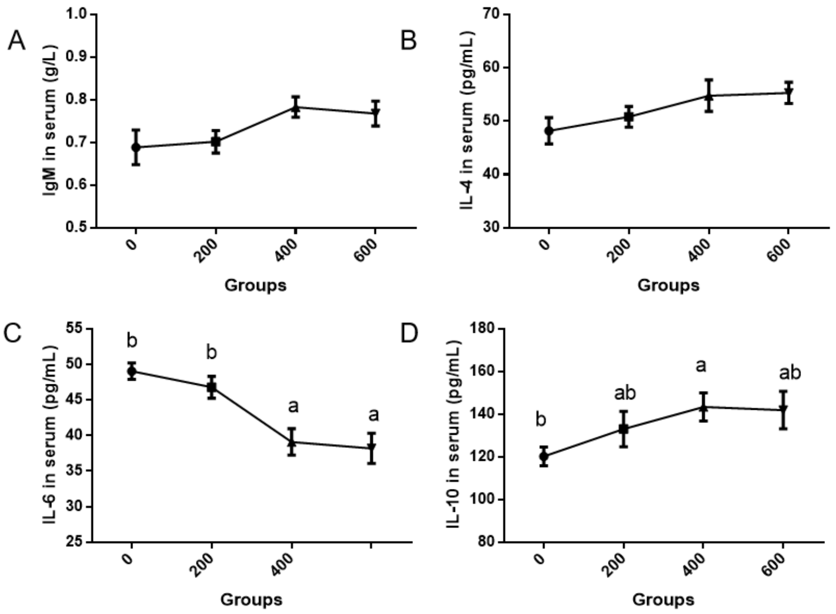

Note: Data represent the means and standard error of mean of 6 replicate cages (n = 6). IgM, immunoglobulin M; IL-4, interleukin-4; IL-6, interleukin-6; IL-10, interleukin-10. Groups: The chickens were administered basal diets containing 0 mg/Kg TCE (control) or 200, 400, or 600 mg/Kg TCE (TCE-supplemented groups). a,b: Means with different letter within the same graph mean significant difference at $p < 0.05$.

Figure S3. Significant effects of *Terminalia chebula* extract (TCE) on the immune function in serum of yellow-feathered broilers on day 56.

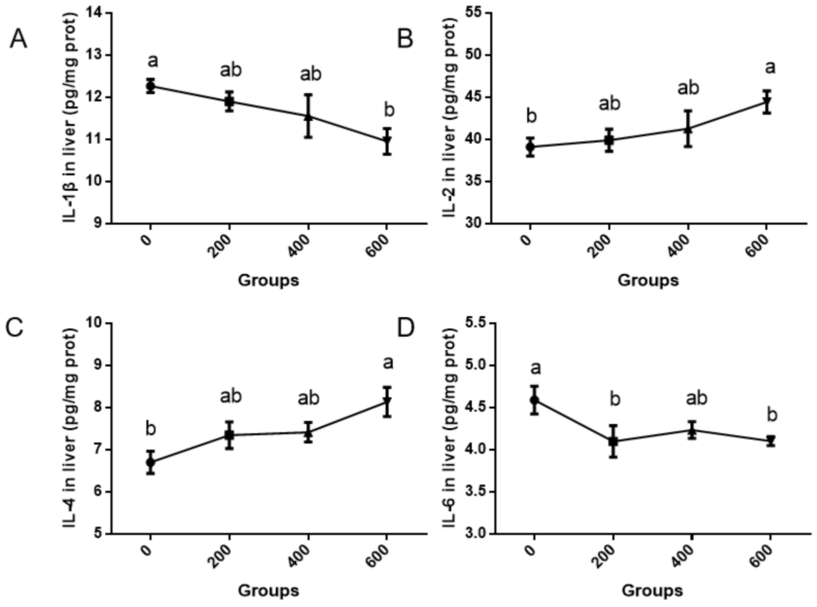

Note: Data represent the means and standard error of mean of 6 replicate cages (n = 6). IL-1β, interleukin-1β; IL-2, interleukin-2; IL-4, interleukin-4; IL-6, interleukin-6. Groups: The chickens were administered basal diets containing 0 mg/Kg TCE (control) or 200, 400, or 600 mg/Kg TCE (TCE-supplemented groups). a,b: Means with different letter within the same graph mean significant difference at $p < 0.05$.

Figure S4. Significant effects of *Terminalia chebula* extract (TCE) on the immune function in liver of yellow-feathered broilers on day 56.

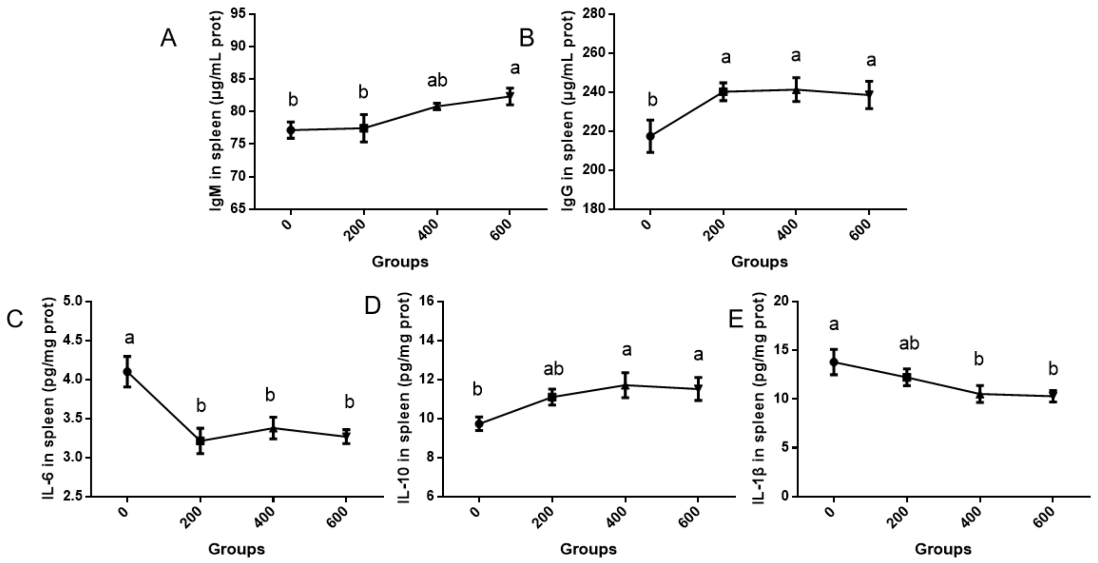

Note: Data represent the means and standard error of mean of 6 replicate cages (n = 6). IgG, immunoglobulin G; IgM, immunoglobulin M; IL-1β, interleukin-1β; IL-6, interleukin-6; IL-10, interleukin-10. Groups: The chickens were administered basal diets containing 0 mg/Kg TCE (control) or 200, 400, or 600 mg/Kg TCE (TCE-supplemented groups). a,b: Means with different letter within the same graph mean significant difference at $p < 0.05$.

Figure S5. Significant effects of *Terminalia chebula* extract (TCE) on the immune function in spleen of yellow-feathered broilers on day 56.

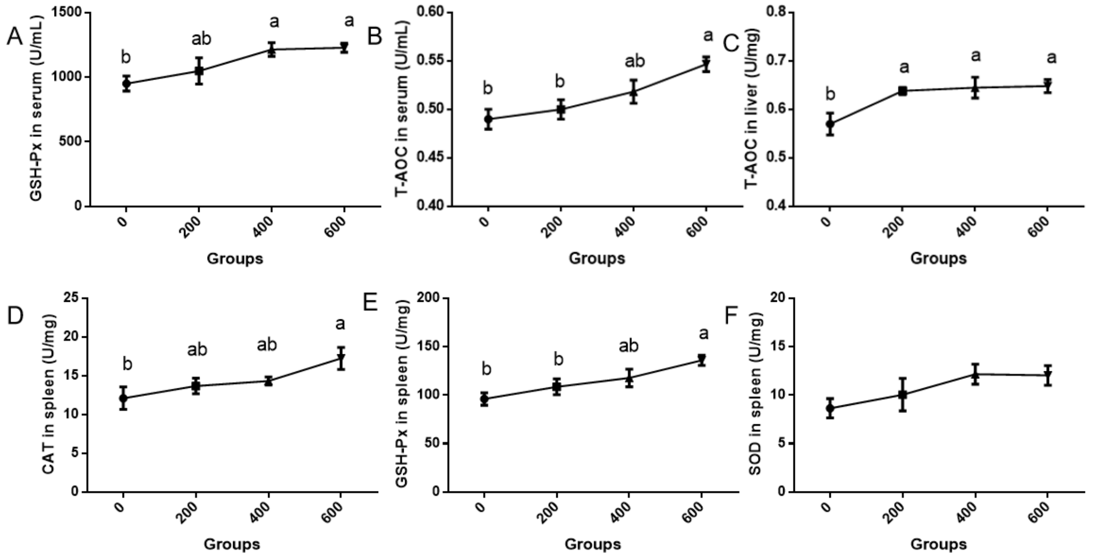

Note: Data represent the means and standard error of mean of 6 replicate cages (n = 6). CAT, catalase; GSH-Px, glutathione peroxidase; T-AOC, total antioxidant capacity; MDA, malondialdehyde; SOD, superoxide dismutase. Groups: The chickens were administered basal diets containing 0 mg/Kg TCE (control) or 200, 400, or 600 mg/Kg TCE (TCE-supplemented groups). a,b: Means with different letter within the same graph mean significant difference at $p < 0.05$.

Figure S6. Significant effects of *Terminalia chebula* extract (TCE) on the antioxidant activity of yellow-feathered broilers on day 56.

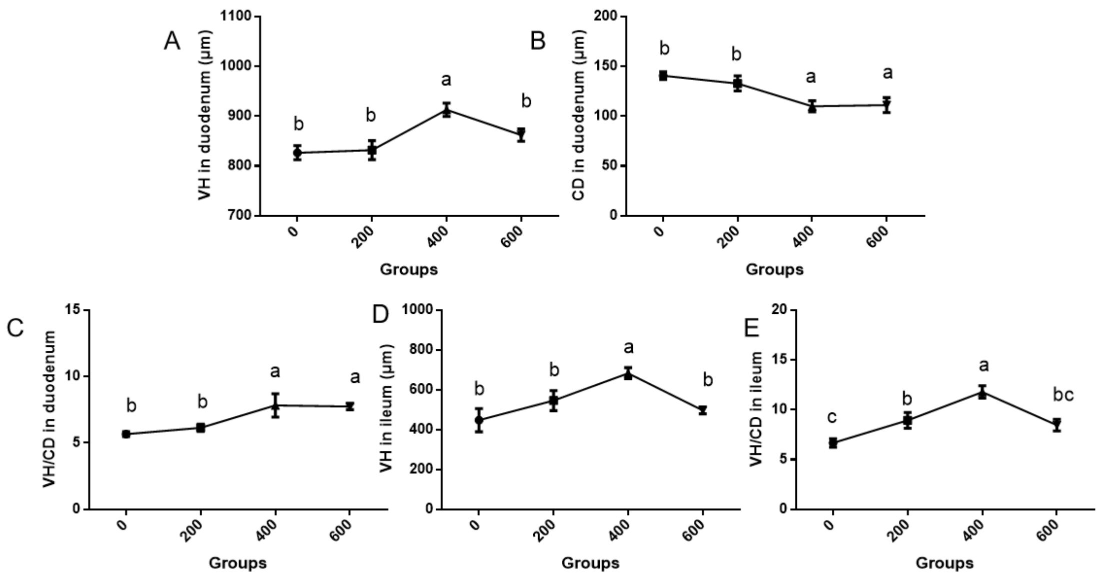

Note: Data represent the means and standard error of mean of 6 replicate cages (n = 6). VH, villus height; CD, crypt depth; VH/CD, the ratio of villus height to crypt depth. Groups: The chickens were administered basal diets containing 0 mg/Kg TCE (control) or 200, 400, or 600 mg/Kg TCE (TCE-supplemented groups). a,b,c: Means with different letter within the same graph mean significant difference at $p < 0.05$.

Figure S7. Significant effects of *Terminalia chebula* extract (TCE) on the intestinal morphology of yellow-feathered broilers on day 56.

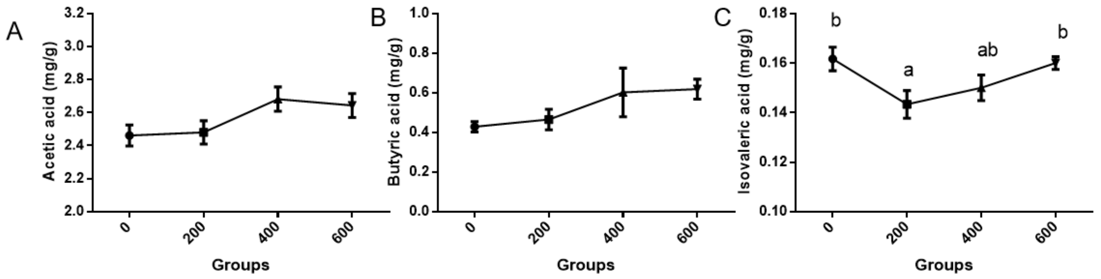

Note: Data represent the means and standard error of mean of 6 replicate cages (n = 6). Groups: The chickens were administered basal diets containing 0 mg/Kg TCE (control) or 200, 400, or 600 mg/Kg TCE (TCE-supplemented groups). a,b: Means with different letter within the same graph mean significant difference at $p < 0.05$.

Figure S8. Significant effects of *Terminalia chebula* extract (TCE) on the short-chain fatty acids of yellow-feathered broilers on day 56.
